# Understanding the Catalytic Active Sites of Crystalline CoSb_*x*_O_*y*_ for Electrochemical Chlorine Evolution

**DOI:** 10.1021/acsami.3c05016

**Published:** 2023-08-18

**Authors:** Heng Dong, Xiaohan Shao, Shane Hancox, Sean T. McBeath, William A. Tarpeh, Michael R. Hoffmann

**Affiliations:** † Linde Laboratories, California Institute of Technology, Pasadena, California 91125, United States; ‡ Department of Civil and Environmental Engineering, Stanford University, Stanford, California 94305, United States; § Department of Civil and Environmental Engineering, University of Massachusetts Amherst, Amherst, Massachusetts 01003, United States; ∥ Department of Chemical Engineering, Stanford University, Stanford, California 94305, United States

**Keywords:** chlorine evolution reaction, electrocatalysis, electrochemical oxidation, electrolysis, scanning electrochemical microscopy, water treatment

## Abstract

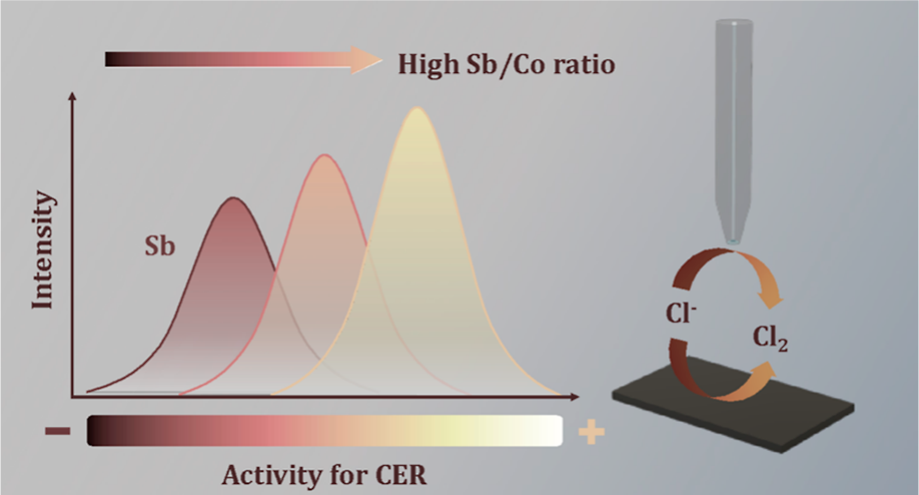

The chlorine evolution reaction (CER) is a key reaction in electrochemical oxidation (EO) of water treatment. Conventional anodes based on platinum group metals can be prohibitively expensive, which hinders further application of EO systems. Crystalline cobalt antimonate (CoSb_*x*_O_*y*_) was recently identified as a promising alternative to conventional anodes due to its high catalytic activity and stability in acidic media. However, its catalytic sites and reaction mechanism have not yet been elucidated. This study sheds light on the catalytically active sites in crystalline CoSb_*x*_O_*y*_ anodes by using scanning electrochemical microscopy to compare the CER catalytic activities of a series of anode samples with different bulk Sb/Co ratios (from 1.43 to 2.80). The results showed that Sb sites served as more active catalytic sites than the Co sites. The varied Sb/Co ratios were also linked with slightly different electronic states of each element, leading to different CER selectivities in 30 mM chloride solutions under 10 mA cm^–2^ current density. The high activity of Sb sites toward the CER highlighted the significance of the electronic polarization that changed the oxidation states of Co and Sb.

## 1 Introduction

Waterborne diseases remain threats to at least one-third of the world population due to the lack of effective sanitation and drinking water provision.^1^ Especially in low- and middle-income countries, prohibitive costs and rapid urbanization limit the application of conventional centralized water treatment and sanitation systems.^2,3^ At current rates of proliferation, only 36% of African and 44% of Asian populations will have access to sewer systems by 2050,^3^ indicating the slow implementation of wastewater treatment systems. Therefore, the development of innovative water treatment solutions, including on-site alternatives to centralized treatment, needs to be addressed to ensure universal sanitation access. Electrochemical oxidation (EO) systems provide a decentralized route for onsite sanitation and water reuse^4^ and have been successfully deployed in China, India, and South Africa.^5,6^ As a core process in EO systems, the chlorine evolution reaction (CER) generates free chlorine (FC) electrochemically at the anode. Electro-generated FC can remove organic contaminants and ammonia and inactivate pathogens,^7,8^ all of which help improve the quality of drinking water and sanitation discharges.^9^

Several characteristics of CER anode materials affect their activity,^10–12^ selectivity,^13–15^ and stability,^16–18^ and therefore the overall performance of EO systems. Dimensionally stable anodes (DSAs), consisting of a Ti base and platinum group metal (PGM) oxides as the active coating layer, have been widely employed for CER catalysis in electrochemical wastewater treatment due to their high activity.^19–22^ However, their larger-scale applications have been limited because of the high price of PGM oxides such as RuO_2_ and IrO_2_ and their long-term instability due to PGM dissolution in acidic media.^23^ To address the challenges of DSAs, several studies have investigated electrocatalysts without noble metals for the CER.^24–31^ For example, Co_3_O_4_ nanobelt array electrodes were synthesized by hydrothermal methods and showed comparable catalytic CER activity to that of RuO_2._^26^ The high activity of the Co_3_O_4_ nanobelt electrodes was attributed to their high surface area. Co–N–C nanoclusters have also been synthesized by pyrolyzing a Co–oxime complex CER catalyst that exhibits higher activity than the RuO_2_/TiO_2_ electrode in 0.4 M HCl solution.^27^ However, Co oxides are prone to dissolution in acidic conditions,^32^ which leads to a short lifetime of the electrodes. On the other hand, PbO_2_ has also been used for CER catalysis but is limited by high overpotential and the reductive leaching of Pb^2+^ ions under open-circuit conditions.^28–31^ Therefore, the search for low-cost, high catalytic efficiency CER electrodes is still ongoing.

In recent years, metal antimonates have been considered as promising alternatives for PGM-based electrodes due to their high catalytic activity, excellent stability, and relatively low price (price difference 5000–10000×).^33–37^ Crystalline CoSb_2_O_*x*_ anodes exhibited comparable CER catalytic activity to the state-of-art RuO_2_–TiO_2_ anodes and even better stability under anodic conditions.^33^ Because Sb-doped Co oxides have different crystalline structures and active site compositions in comparison to Co oxides, the catalytic mechanisms of crystalline CoSb_2_O_*x*_ remain unclear. Attempts have been made to elucidate the catalytic mechanism of amorphous CoSb_2_O_*x*_ for the oxygen evolution reaction (OER), where the oxygen vacancy and Co(IV) species were identified to be the primary active sites.^38^ However, the active sites for the CER have not been determined for their crystalline counterparts.

The objective of this study was to identify the catalytically active sites for crystalline CoSb_*x*_O_*y*_ for the CER and understand the effect of Sb/Co ratios on EO performance through material and electrochemical characterization. Specifically, we aimed to (1) fabricate crystalline CoSb_*x*_O_*y*_ anodes with various Sb/Co ratios; (2) understand the differences in their material properties (e.g., bulk and surface stoichiometries) via energy-dispersive spectrometry (EDS) and X-ray photoelectron spectroscopy (XPS); and (3) characterize and compare the CER catalytic activities of Co and Sb sites in crystalline CoSb_*x*_O_*y*_ anodes through bulk and near-electrode electrochemical measurements such as specific activity, electrochemical surface area (ECSA), and scanning electrochemical microscopy (SECM). This study also provides insights into the selectivity of CoSb_*x*_O_*y*_ anodes for the CER over the OER in dilute chloride solution, which complements the catalytic activity study with an improved understanding of how crystalline CoSb_*x*_O_*y*_ anodes enable the CER in EO wastewater treatment. Results from this study can also help improve the performance and implementation of EO systems, motivate innovations in decentralized water treatment processes, and eventually contribute to universal access to clean water and sanitation.

## 2 Materials and Methods

### 2.1 Electrode Fabrication

To prepare for electrodeposition, Ti plates (1 × 2 cm) were degreased with acetone and etched in boiling 10 wt % oxalic acid solution for an hour to remove the native oxide layer. An undivided three-electrode electrochemical reactor was used for electrodeposition with a commercial RuO_2_–IrO_2_–TiO_2_/Ti counter electrode and a saturated calomel reference electrode (*E* = +0.244 V vs SHE at 25 °C). Mixed films of Co(OH)_2_ and metallic Sb were electrochemically deposited onto Ti plates as the precursor for CoSb_*x*_O_*y*_, which was used as the working electrode. The electrolyte consisted of 430 mM CoCl_2_, 115 mM K_2_Sb_2_ (C_4_H_2_O_6_)_2_, and 400 mM KNO_3_, as modified from a previous study.^39^ Films with different Sb/Co ratios were reductively deposited at different applied potentials, and the total charge passed was controlled at 1 mA h for the deposition process. At more negative potentials, the Faradaic efficiency (FE) for the hydrogen evolution reaction with respect to the reduction of K_2_Sb_2_ (C_4_H_2_O_6_)_2_ into metallic Sb increased during electrodeposition, which caused the differences in the bulk Sb/Co ratio. Afterward, the deposited films were rinsed with nanopure water (resistivity: 18.2 mΩ·cm), dried under ambient air, and then annealed at 600 °C for 6 h at a ramping rate of 10 °C min^–1^ to produce the crystalline CoSb_*x*_O_*y*_ films. Comparative CER experiments were also conducted using a commercial IrO_2_ electrode (De Nora Water Technologies) with the same active surface area as that of the fabricated electrodes.

### 2.2 Material Characterization

The morphology and bulk elemental composition of the electrodes were examined by using a ZEISS 1550VP field emission scanning electron microscope (Oberkochen, Germany) equipped with an Oxford X-Max SDD energy-dispersive X-ray spectrometer. The crystalline structures of the samples were characterized with a Rigaku SmartLab X-ray diffractometer (Tokyo, Japan) with a Cu Kα radiation (λ = 1.5418 Å) source. The surface stoichiometry and electronic states of the elements were examined with a Surface Science M-Probe XPS system with an Al Kα monochromatic X-ray source. The pressure in the measurement chamber was controlled at ∼1 × 10^–9^ Torr during measurement. The data were analyzed using CasaXPS software, and a Shirley background was used to quantify the XPS peak areas.

### 2.3 Electrochemical measurements

#### 2.3.1 Electrochemical Characterization in Bulk Solution

The ECSA was estimated from the electrochemical double-layer capacitance (*C*_DL_) of crystalline CoSb_*x*_O_*y*_ in 1 M H_2_SO_4_ (specific capacitance *C*_s_ = 0.035 mF cm^–2^) based on a previously reported method to facilitate comparison of activities across catalysts.^40^ To determine *C*_DL_, a non-Faradaic capacitive current was obtained from cyclic voltammetry (CV) at different scan rates (BioLogic VSP-300, Warminster, France). A 100 mV wide potential window centering the open-circuit potential (OCP) was adopted for CV measurements at scan rates of 5, 10, 25, 50, 100, 200, 400, and 800 mV s^–1^, respectively. The measured current at the OCP was plotted against the scan rate to calculate C_DL_, which was then converted into the ECSA according to eq 1
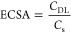
1

Specific activities denoted the catalytic current densities normalized to ECSAs, and they were used to characterize the catalytic activities of the anodes. Specifically, linear sweep voltammetry (LSV) was performed at a scan rate of 10 mV s^–1^ in an undivided three-electrode system with a Ti plate (2 × 3 cm) counter electrode and a saturated calomel reference electrode. Voltammograms were taken in both a 5 M NaCl solution and 0.1 M H_2_SO_4_ solution to examine the catalytic activities for the CER and the OER, respectively.

Catalytic selectivity toward the CER vs the OER was examined by measuring the aqueous FC concentration in 30 mM NaCl solution and calculating the FE for different fabricated electrodes. A 20 mA constant current condition was adopted to ensure a geometric current density of 10 mA cm^–2^ (electrode geometric surface area 2 cm^2^). The FC concentration was measured by using DPD (*N*,*N*-diethyl-*p*-phenylenediamine) reagent (Hach methods 10101 and 10102).

#### 2.3.2 Scanning Electrochemical Microscopy in the Diffusion Layer

SECM experiments were performed in a customized electrochemical reactor with a five-electrode configuration (2 working electrodes, 2 counter electrodes, and 1 reference electrode; setup shown in Figure S1a) with 5 M NaCl electrolyte to maximize the FE and avoid chloride concentration as the limiting factor.^41,42^ The stage position and electrochemical measurements were controlled and recorded by a BioLogic M470 scanning electrochemical workstation coupled to a BioLogic SP-300 potentiostat (Warminster, France). In addition to the fabricated crystalline CoSb_*x*_O_*y*_ film electrodes, a Ti electrode coated with Ir/Ta MMO (mixed metal oxide; Titan Metal Fabricators, Camarillo, CA) was used to establish the baseline for high CER activity because it is considered a suitable CER catalyst.^43^ A commercial ultramicroelectrode (UME; BioLogic Sciences Instruments, Warminster, France) with a 10 μm Pt tip was used as another working electrode, which was freshly polished with sandpaper prior to use. A glassy carbon (GC) microelectrode and a stainless-steel sheet were used as the counter electrode for the tip and the fabricated electrodes, respectively. A Ag/AgCl electrode (+0.21 V vs SHE) was used as the reference electrode for both working–counter electrode pairs (substrate and tip) to facilitate separate control of applied potential for each pair.

Bulk CV measurements were conducted on both sets of electrodes (Figure S2) to determine the corresponding potentials for the CER on each working electrode. Another consideration for choosing the potential was to minimize chlorine gas bubble formation to avoid misrepresentative current measurement in SECM due to the coverage of bubbles on either one of the working electrodes. Specifically, +1.4 V vs Ag/AgCl was applied to the tip and +1.3 V vs Ag/AgCl was applied to the sample, which were the minimal viable potentials for the CER for the working electrodes when both were biased.

Intermittent contact scanning electrode microscopy (IC-SECM) was employed to decouple the contribution to the measured current from the electrode topography and electrochemical activity (Figures S3 and S4). SECM experiments were conducted when the UME tip was 5 μm above (within the diffusion layer) the surface of the fabricated electrodes. The tip moved with a step size of 10 μm in both the *x* and *y* directions (across the substrate surface) at a scan speed of 20 μm s^–1^. For each fabricated electrode, two 50 × 50 μm area maps were generated: one with only the tip being biased (background) and another with both the tip and the fabricated electrode being biased (redox competition) to catalyze the CER (Figure S1b).^44^ The fabricated electrode activity was determined by subtracting the tip current under redox competition from the background tip current. The difference in tip current resulted from the fabricated electrode competing for the redox species (chloride in this study), and a larger percentage change in current indicated higher activity toward the CER of the fabricated electrode.

## 3 Results and Discussion

### 3.1 Material Characterization

#### 3.1.1 Bulk and Surface Sb/Co Ratios

**Figure 1:**
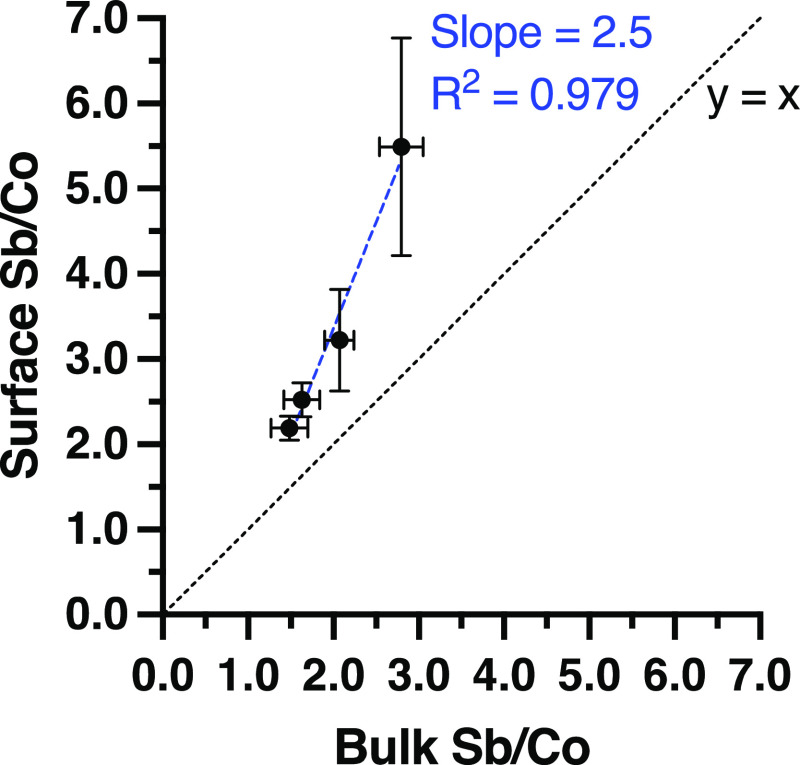
Comparison of surface and bulk Sb/Co ratios. The surface Sb/Co ratios were obtained by XPS, while the bulk Sb/Co ratios were obtained by EDS. Error bars represent ±1 standard deviation (*n* = 3).

Electrodes with different bulk Sb/Co ratios were produced by annealing Co(OH)_2_/Sb films that were electrodeposited at different potentials. Based on the EDS spectra, the bulk Sb/Co ratio generally decreased with more negative applied potentials (Figure S5). From −0.85 to −1.05 V, the bulk Sb/Co ratios exhibit a nearly 2-fold decrease from 2.80 to 1.49. Compared with bulk Sb/Co ratios, the surface Sb/Co ratios were observed to be considerably higher (Figure 1, slope of the line of the best fit = 2.5). Considering the instability of Co species (i.e., dissolution) in acidic media,^32^ the enriched Sb at the surface might account for the activity and acid stability of CoSb_*x*_O_*y*_ catalysts. This role is further motivated by the observed Sb activity for the oxygen reduction reaction (ORR), along with the stability of antimonate frameworks for the OER and ORR.^45,46^ A similar effect was reported in RuO_2_–TiO_2_ anodes, suggesting that after being electronically tuned, TiO_2_ acted as a more active catalytic site than RuO_2_ based on both experimental and computational evidence.^47–49^ Turning the “stabilizer elements” (e.g., Ti in RuO_2_–TiO_2_ and Sb in CoSb_*x*_O_*y*_) into active catalytic sites provides a promising approach to enhance the electrode performance by coupling the catalytic activity with stability.

#### 3.1.2 Surface Morphology

A mud crack morphology was observed on CoSb_*x*_O_*y*_ samples (Figure S6); these cracks were formed under large tensile stress during the process of annealing electrodeposited Co(OH)_2_/Sb films at different potentials.^50–52^ The width of the cracks was generally larger for lower bulk Sb/Co ratios, ranging from approximately 0.5 to 3.0 μm across Sb/Co ratios from 2.80 to 1.43. EDS maps showed a generally homogeneous distribution of Co and Sb on the sample planes (Figure S7), suggesting that the aggregates of particle spheres were likely not due to one single element. However, it has been reported that cobalt oxide could lead to the formation of aggregates on the coating surface.^53^

#### 3.1.3 Crystalline Structure and Electronic States

The samples generally maintained the rutile structure of CoSb_*x*_O_*y*_ across different Sb/Co ratios (Figure S8a), while the crystallinity varied substantially with the stoichiometry. Specifically, no significant change in the X-ray diffraction (XRD) pattern was observed as the stoichiometry shifted from a Sb/Co ratio of 2.07 to 2.80. While amorphousness was observed at lower Sb/Co ratios (i.e., no XRD signal), no distinct cobalt oxide phase was observed, suggesting that excess Co and Sb were doped into the rutile lattices. However, the possible existence of amorphous CoO_*x*_ and SbO_*x*_ cannot be excluded.

Because the catalytic CER takes place at the electrode surface, the surface crystallinity of the samples is especially important for facilitating catalytic activity. Grazing incidence XRD (GIXRD) showed that the rutile structure was maintained at the catalyst surface (Figure S8b). The intensities of the GIXRD spectra were generally lower than the bulk XRD spectra because of the small incident angle (4° in this study) and, therefore, the small survey depth. Consistent with the bulk XRD results, Sb-rich samples exhibited better rutile crystallinity, while Co-rich samples were more amorphous. From a search match analysis (see XRD methods in the Supporting Information), CoSb_*x*_O_*y*_ was identified as the primary plausible crystalline phase present for all deposition potentials (−0.85, −0.90, −0.95, −1.00, and −1.05 V vs SCE). A detailed overview of XRD peak analysis, which was generated from a separate set of electrode samples with identical composition, is summarized in Table S1 and Figure S9 in the Supporting Information.

**Figure 2:**
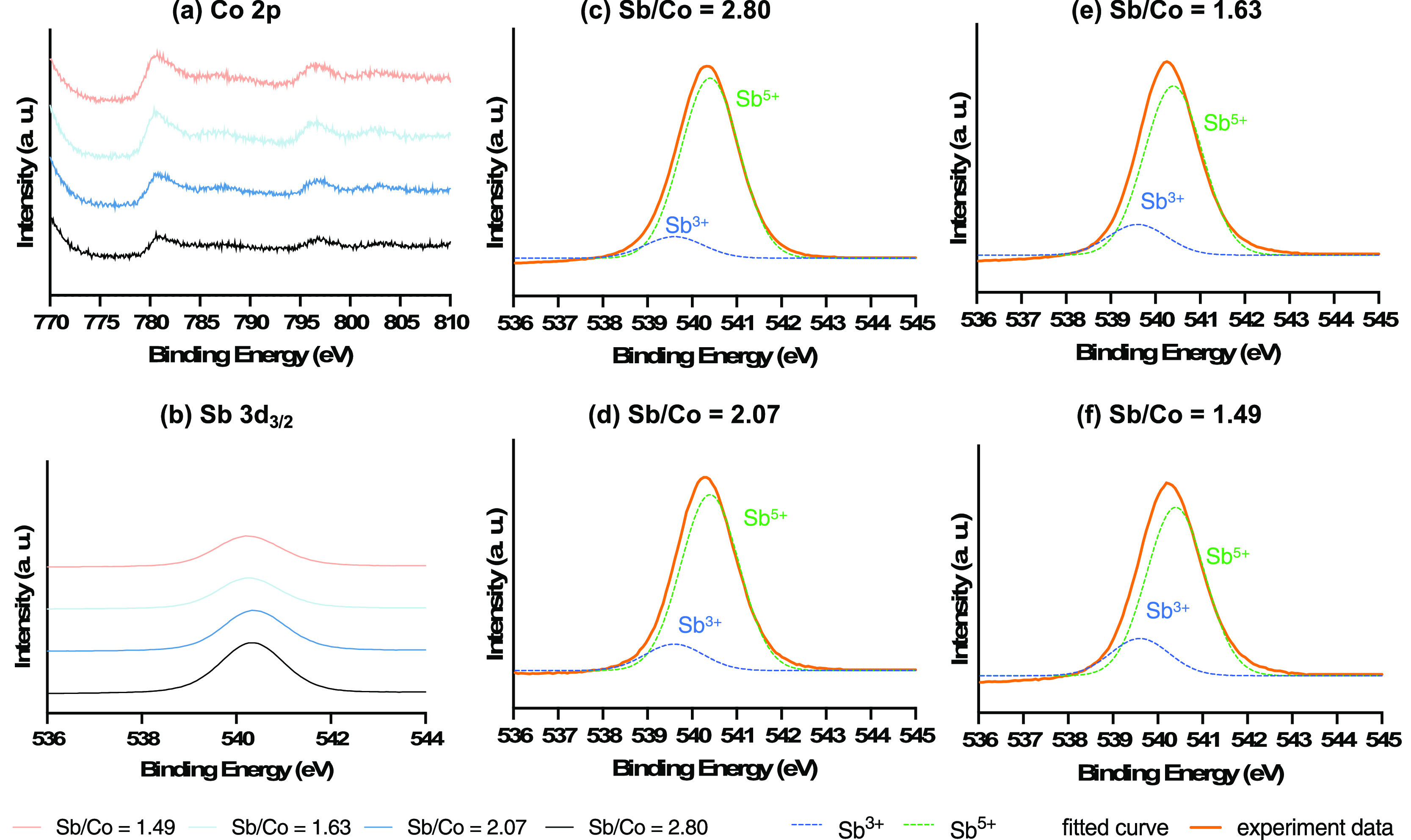
XPS spectra of (a) Co 2p and (b) Sb 3d_3/2_ for various cobalt antimonate samples with different Sb/Co ratios and (c–f) deconvolution of Sb 3d_3/2_ peaks.

The electronic states of Co and Sb were examined by XPS (Figure 2). Co 2p_1/2_ and Co 2p_3/2_ peaks were in the ranges of 780.6–780.7 and 796.6–796.7 eV, respectively. The Co 2p_1/2_ peak position aligned with previously reported values for CoO, while the 2p_3/2_ peak position was slightly more positive than other reports.^54,55^ The Sb 3d_3/2_ peak was observed in the range of 540.0–540.4 eV,^56,57^ which spanned the previously reported binding energies for both Sb_2_O_3_ and Sb_2_O_5_. The Sb 3d_5/2_ peak was excluded from this analysis due to considerable overlap with O 1s spectra.

At lower Sb/Co ratios, the Co 2p peak positions shifted systematically in the more positive direction, while the Sb peaks shifted toward the more negative direction. Deconvolution of the Sb 3d_3/2_ peak was conducted using Gaussian lines to indicate the presence and the change of oxidation states of Sb (center of the peak: 539.6 eV for Sb^3+^ and 540.4 eV for Sb^5+^) in the crystalline CoSb_*x*_O_*y*_ samples (Figure 2).^58–61^ As the Sb/Co ratios increased, the presence of Sb^5+^ became more abundant in the sample, the Sb^3+^/Sb^5+^ ratios decreased, and the corresponding binding energy of the Sb 3d_3/2_ peak became higher. For example, from the samples with an Sb/Co ratio of 1.49 to 2.80, the percentage of Sb^5+^ increased from 81.9 to 89.3%, the Sb^3+^/Sb^5+^ ratios decreased from 0.22 to 0.12, and the Sb 3d_3/2_ peaks shifted from 540.2 to 540.3 eV (Figure S10). These shifts across samples with different Sb/Co ratios indicated a potential electronic polarization between Co and Sb in the cobalt antimonate samples such that Sb in the sample was slightly more oxidized at a higher Sb/Co ratio.^47,62^

Considering the nominal valences of Co (+2) and Sb (+5) in CoSb_*x*_O_*y*_, it was worthwhile to note that the XPS peak positions of neither Co nor Sb directly aligned with those of the individual metal oxides (CoO and Sb_2_O_5_). This result indicated that both elements had altered electronic states compared with their individual metal oxides, and this significant electronic interaction could explain the catalytic capability of crystalline CoSb_*x*_O_*y*_.

### 3.2 Electrochemical Characterization

#### 3.2.1 Bulk Electrochemical Measurement

To evaluate the catalytic activity of the cobalt antimonate samples for the CER, electrochemical characterization techniques were conducted in both the bulk and the diffusion layers. Shown in the specific activities results (Figure S11a,b), which denoted the catalytic current densities normalized to the ECSAs (Figure S11c), the samples with smaller ECSAs (Sb/Co = 2.80 and Sb/Co = 1.49) exhibited substantially higher specific activities than those with higher ECSAs (Sb/Co = 2.07 and Sb/Co = 1.63) for both the CER (tested in 5 M NaCl) and OER (tested in 0.1 M H_2_SO_4_). This discrepancy can be traced to bubble formation at the sample surface under a higher potential, which blocked the mass transport of both reactants and products during the LSV measurements. The reason why the samples with smaller ECSAs exhibited higher specific activities was that fewer bubbles were present at the sample surface, and therefore, the specific activities generally followed the reverse order of ECSAs. One exception was observed for the most Co-rich sample (Sb/Co = 1.49), which had substantially larger cracks (Figure S6) than the most Sb-rich sample (Sb/Co = 2.80). This mud crack morphology has been shown to be favorable for chlorine and oxygen bubble release during the CER and OER.^42,50,63^ and explains why the Sb/Co = 1.49 sample exhibited enhanced specific activity compared to other samples.

#### 3.2.2 Diffusion Layer Electrochemical Measurement Using SECM

**Figure 3:**
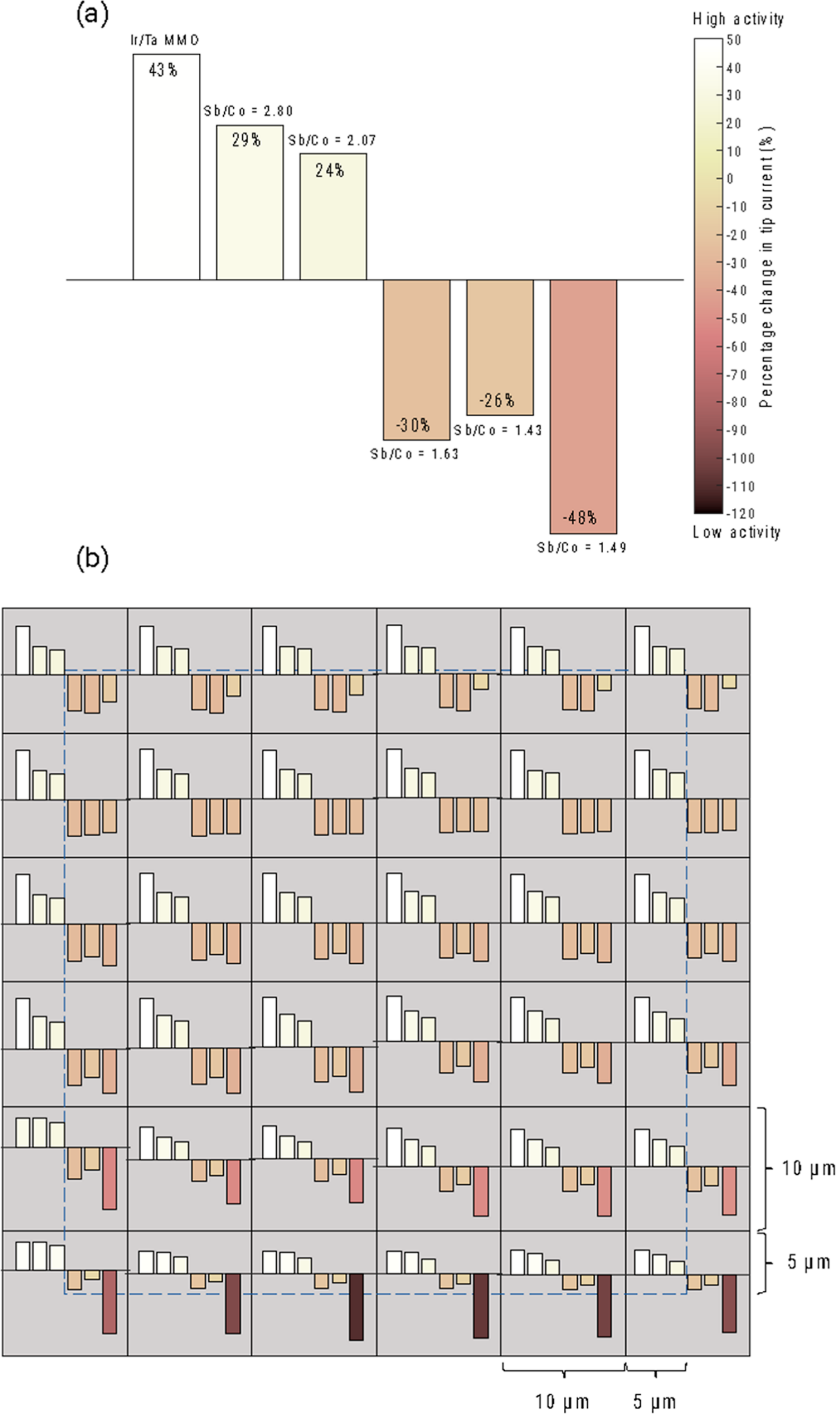
(a) Average percentage changes in tip current for cobalt antimonates with different Sb/Co ratios. (b) Each block represents a 10 × 10 μm scan area on the electrode, and the area of interest (50 × 50 μm) is within the dashed line (shaded in blue). Measurements were taken at the center of each 10 × 10 μm block. Activities of the electrodes toward the CER are represented by the bar graphs inside each block. From left to right, the bars represent the activities of Ir/Ta MMO, Sb/Co 2.80, 2.07, 1.63, 1.43, and 1.49, respectively [the same order as that in subfigure (a)]. The percentage change in tip current was calculated between two conditions: at the OCP and biased at 1.3 V vs Ag/AgCl reference electrode (see Figure S12 for these two scenarios). The average percentage change across the entire sample area is indicated below the Sb/Co ratio on each subfigure. A higher percentage change in tip current correlates to higher activity toward the CER of the electrode.

Conventional techniques, such as LSV, were not the best tool to objectively compare catalytic performance across samples due to sample topology differences and mass transport limitations caused by oxygen and chlorine bubble generation. Therefore, IC-SECM and SECM were used in this study to semiquantitatively compare the catalytic activities of the cobalt antimonate samples for the CER under the condition for minimal bubble generation. In general, the catalytic activities of the samples increased with the Sb content (Figure 3). However, the activities of the samples with Sb/Co ratios of 2.07 and 2.80 were relatively close, indicating that the catalytic activities of the CER were insensitive to the stoichiometry in a certain stoichiometry window.

From the SECM results, Sb sites in crystalline CoSb_*x*_O_*y*_ exhibited higher catalytic activity for the CER than Co sites (i.e., higher activity with a higher Sb/Co ratio). Compared to a recent report that identified amorphous CoSb_*x*_O_*y*_ with Co sites that are more active catalytic sites than the Sb sites,^38^ the conclusion in our study differs and suggests that the mechanism for amorphous CoSb_*x*_O_*y*_ does not apply to its crystalline counterparts. The activity of the Sb sites may originate from the electronic interaction between Co and Sb in crystalline CoSb_*x*_O_*y*_, which was confirmed by XPS spectra (Figure 2). Similar effects were not observed in amorphous systems because Co and Sb in such systems do not have long-range-ordered atomic arrangements, which resulted in the lack of influence of Co on Sb across the bulk electrode. Considering the surface segregation of Sb in CoSb_*x*_O_*y*_ and the leaching of Co under acidic pH conditions, the top Sb layer in the crystalline CoSb_*x*_O_*y*_ served as an active, stable CER catalyst. This conclusion aligns with the proposed role for Sb as a stabilizing structure constituent under acidic conditions.^64^

### 3.3 Selectivity

**Figure 4:**
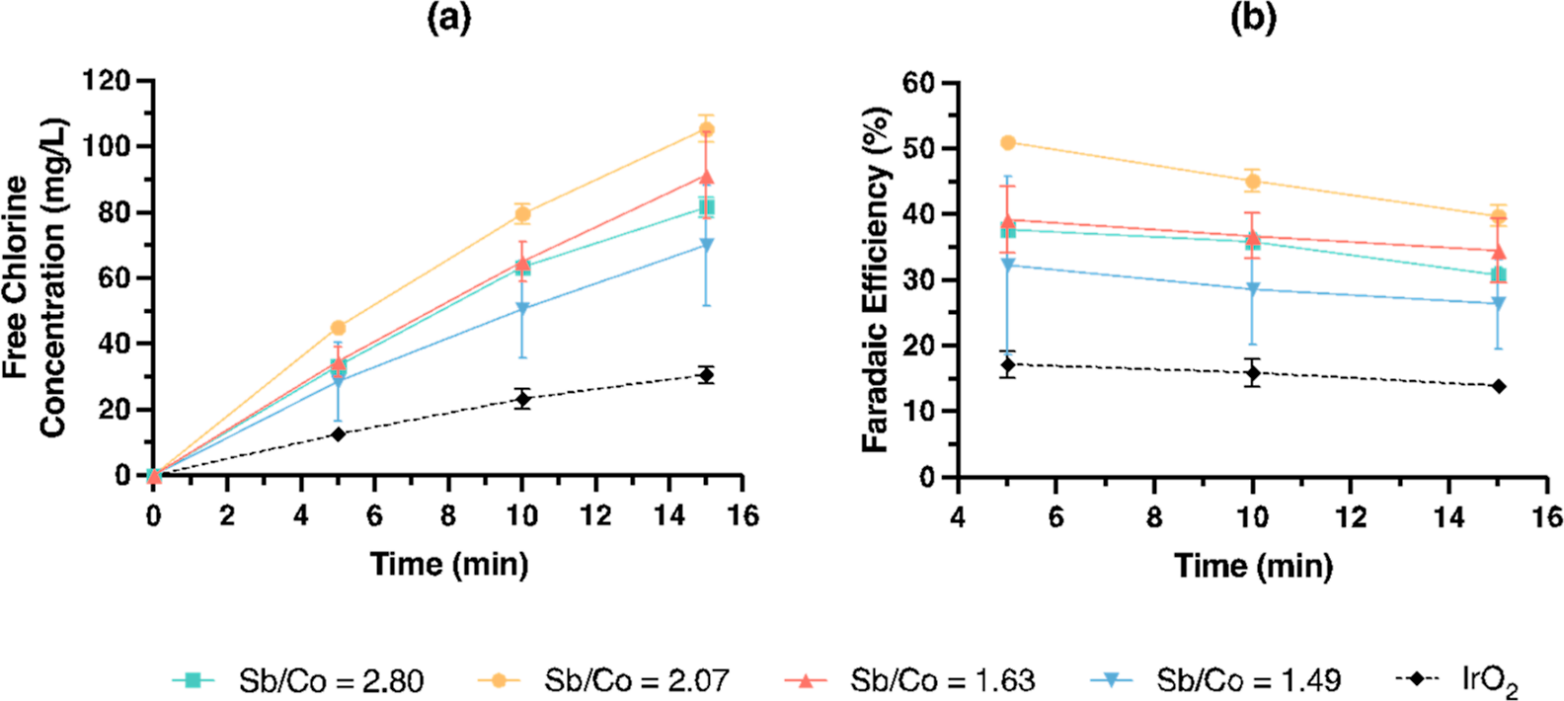
Evolution of (a) FC concentration and (b) FE over time. The electrolyte was 30 mM NaCl solution, and the applied current was 20 mA for all samples. Error bars represent ±1 standard deviation (*n* = 3).

The selectivity of the electrodes for the CER over the OER in dilute chloride solutions was examined by measuring the FC concentration and FE (Figure 4). The change of the CER FE with the stoichiometry was not monotonic. The Sb/Co = 2.07 sample had the highest cumulative CER FE of 40 ± 2% in 15 min, followed by Sb/Co = 1.63 (34 ± 5%), Sb/Co = 2.80 (31 ± 1%), and Sb/Co = 1.49 (26 ± 7%). As a general benchmark test for CER activity, comparative tests were performed by using a commercial IrO_2_ electrode. Under the dilute chloride conditions, CoSb_*x*_O_*y*_ outperformed IrO_2_ in terms of FE toward the CER (Figure 4); the IrO_2_ electrode had an average FE of 15.7% compared to the 29.1–45.3% with the CoSb_*x*_O_*y*_ electrode.

Although the selectivity for the CER over that for the OER is one of the most important indicators for electrochemical water treatment performance, the intertwined mechanisms of the two reactions make it difficult to create electrodes that are highly selective for the CER, especially in dilute chloride solutions. As reported by various computational studies, the CER and OER share important intermediate species [e.g., O^c^ and OH^c^ for RuO_2_ (110), where ^c^ denotes coordinatively unsaturated sites].^65^ Thus, their free energy barriers often change synchronously when the catalyst materials are varied.^65–67^ However, it is still possible to achieve a highly selective CER by lowering the free energy barrier for the CER while raising that for the OER, which requires fine tuning of the adsorption energy of the catalyst surfaces for some of the key intermediates, such as Δ*E*(O^c^).^68^

The selectivity differences of the samples were attributed to the different electronic states of the Co and Sb atoms, as revealed by XPS (Figure 2). Because the electronic states of the elements changed with the electrode stoichiometry, it was expected that the adsorption energy for oxygen- and chlorine-containing intermediate species was also adjusted. The highest selectivity (Sb/Co = 2.07) sample indicated that the electronic states of the Co and Sb were optimal to produce the largest free energy barrier difference between the CER and the OER. This conclusion indicated that varying the stoichiometry in CoSb_*x*_O_*y*_ systems may not only change the ratio of the two catalytic sites but also slightly modify the properties of each catalytic center. This change in selectivity again further addresses the importance of electronic interaction between the metal elements.

## 4 Conclusions

In this study, we proposed that Sb in crystalline CoSb_*x*_O_*y*_ accounted for its CER activity, which was derived from the electronic interaction between Co and Sb. Based on material and electrochemical characterization, this electronic interaction could also be responsible for the observed selectivity between the CER and OER. We also found that the mud crack morphology formed during the annealing process favored the release of bubbles generated from the OER and CER. This unique morphology is subject to further investigation and utilization as a promising strategy to improve the catalytic efficiency of crystalline CoSb_*x*_O_*y*_. The conditions at the electrode–electrolyte interface may also affect the CER, which is subject to further study. For example, SECM can be incorporated to measure the local pH in the diffusion layer. As one pillar to electrocatalyst design (activity, selectivity, and stability), the stability of crystalline CoSb_*x*_O_*y*_ should also be evaluated in extreme conditions (e.g., strongly acidic environment, often < pH 2) and in terms of mechanical durability and adhesion. The stability of the catalyst influences the useable lifetime of electrodes, which is one crucial factor that affects the performance and cost of EO.^69^ As the next step after the material and electrochemical characterization conducted here, we anticipate useful insights from studying crystalline CoSb_*x*_O_*y*_ integrated in electrochemical reactors and translated toward EO applications. To better articulate the importance of crystalline CoSb_*x*_O_*y*_ as a CER catalyst and to advance sanitation and water reuse, future studies should aim to improve the engineering design and integration of crystalline CoSb_*x*_O_*y*_ with EO systems. One effective approach would be the strategic design of crystalline CoSb_*x*_O_*y*_ electrodes with a morphology that better assists the release of bubbles generated during the OER and CER to reduce mass transport limitations. There is also a need for reactor and process engineering that integrates crystalline CoSb_*x*_O_*y*_ anodes with cathode materials (e.g., for dehalogenation and hydrogen evolution), reactor geometry, and operating conditions. Finally, future efforts could focus on understanding crystalline CoSb_*x*_O_*y*_ as an anode under more realistic and complex conditions, including treating various influent streams (e.g., municipal wastewater, reverse osmosis concentrate, graywater, and urine) and integrating EO as a module in various wastewater treatment trains (e.g., onsite sanitation, centralized treatment, and hybrid approaches). Together, these future efforts could accelerate the informed translation efforts of CoSb_*x*_O_*y*_ to well-suited applications that realize its potential role in enhancing sustainable sanitation and drinking water access.

## Supporting Information Available

Setup and redox competition schematic for SECM experiments, CV of the SECM tip and fabricated electrodes, DC and AC response from the SECM piezoelectric positioner, topology of fabricated electrodes, bulk Sb/Co ratio at different potentials, SEM images, EDS mapping, XRD and GIXRD, XPS peak deconvolution, specific activities, ECSA of fabricated electrodes, and explanation on two scenarios of SECM measurements (PDF)
