# Supplementary material for: Understanding the Catalytic Active Sites of Crystalline CoSbxOy for Electrochemical Chlorine Evolution
Source: ACS Appl Mater Interfaces. 2023 Aug 18;15(34):40369–77. doi: 10.1021/acsami.3c05016 (PMC10472335; doi:10.1021/acsami.3c05016)
Supplement: Supplementary file 1 — am3c05016_si_001.pdf [file am3c05016_si_001.pdf]

Supporting Information

Understanding the catalytic active sites of crystalline $CoSb_xO_y$ for electrochemical chlorine evolution

Heng Dong[1#], Xiaohan Shao[2#], Shane Hancox[3], Sean T. McBeath[3], William A. Tarpeh[2, 4*], and Michael R. Hoffmann[1*]

[1] Linde Laboratories, California Institute of Technology, Pasadena, CA, 91125, United States

[2] Department of Civil and Environmental Engineering, Stanford University, Stanford, CA, 94305, United States

[3] Department of Civil and Environmental Engineering, University of Massachusetts, Amherst, MA, 01003, United States

[4] Department of Chemical Engineering, Stanford University, Stanford, CA, 94305, United States

#H.D. and X.S. contributed equally to this paper.

* Corresponding author, Email: wtarpeh@stanford.edu. Address: 443 Via Ortega, Room 387, Stanford CA, 94305, United States. Telephone: (650) 497-1324

Figures: 12

Table: 1

Submitted to: *ACS Applied Materials & Interfaces*

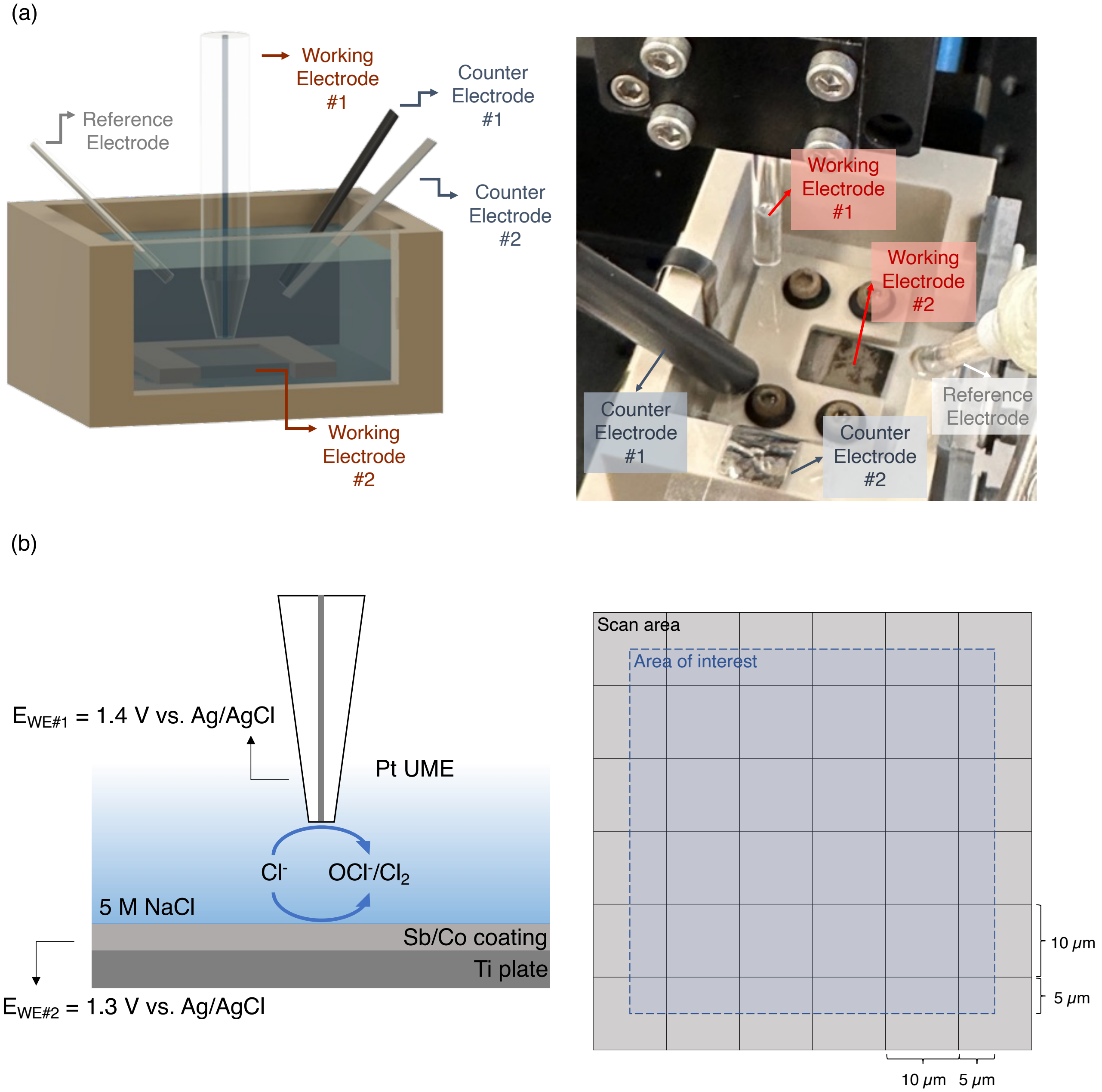

Figure S1. (a) The setup for scanning electrochemical microscopy (SECM) experiments. The reactor was customized and fabricated (materials: PEEK for the main body, and polycarbonate for the window to allow monitoring on the side) with a five-electrode configuration. Working electrode #1 was a commercial platinum ultra-micro electrode (UME; 10 μm diameter; BioLogic Sciences Instruments, Warminster, France). Working electrode #2 was the fabricated crystalline $CoSb_xO_y$ film electrodes, and Ir/Ta mixed metal oxide (MMO; Titan Metal Fabricators, Camarillo, CA). The two counter electrodes were glassy carbon (#1, paired with tip) and stainless steel (#2, paired with either $CoSb_xO_y$ or MMO), respectively.

The reference electrode was Ag/AgCl electrode (+0.21 V vs. SHE). 5 M NaCl electrolyte was used for all SECM experiments. (b) The redox competition schematic for the SECM measurements. Both working electrodes were competing for chloride in the electrolyte. The applied potentials for working electrodes #1 and #2 were 1.4 and 1.3 V vs. Ag/AgCl, respectively, according to the onset potentials for CER from cyclic voltammetry. Each block represents a 10 μm x 10 μm scan area on the electrode, and the area of interest (50 μm x 50 μm) is within the dash line (shaded in blue). Measurements were taken at the center of each 10 μm x 10 μm block.

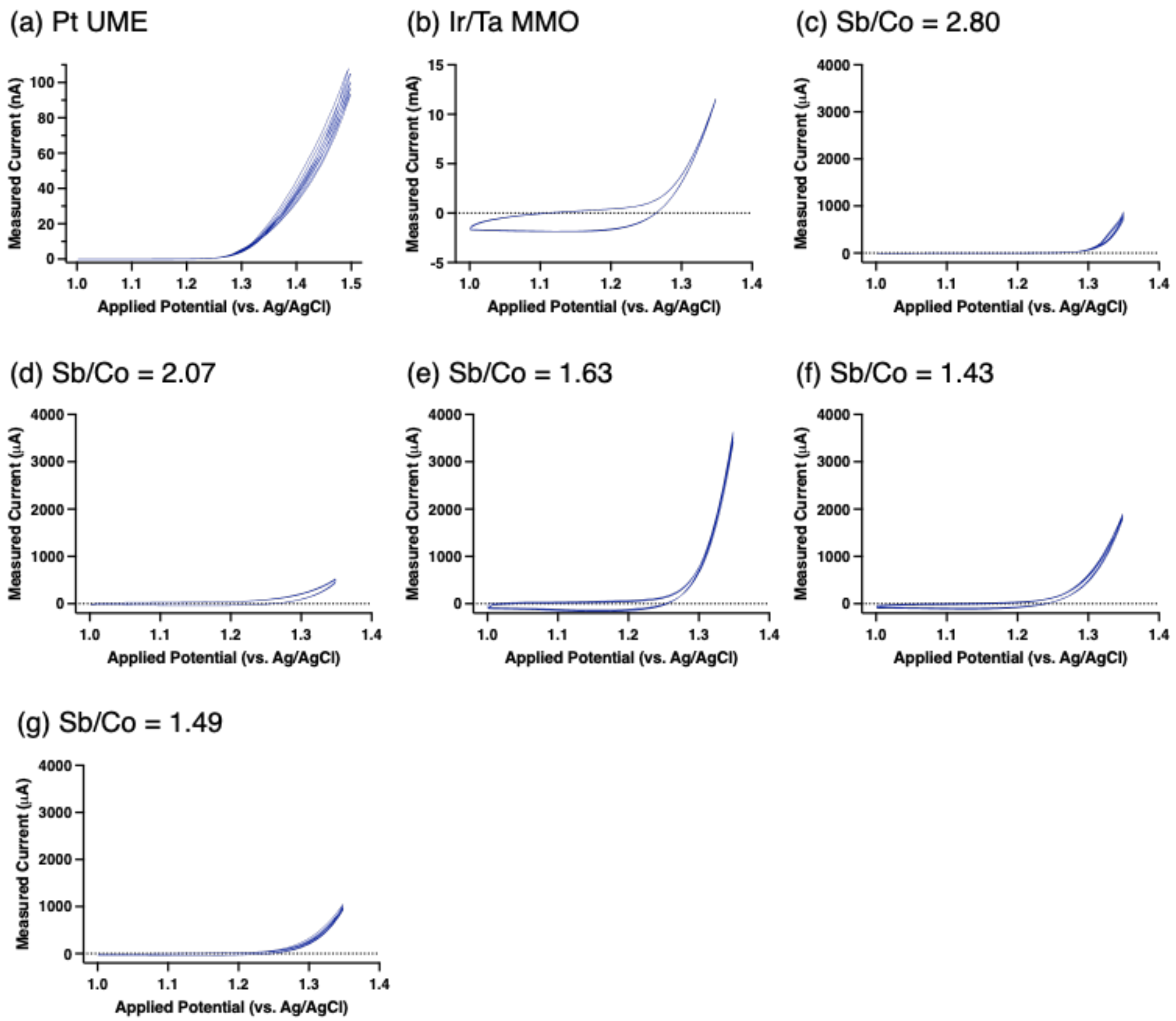

Figure S2. Cyclic voltammograms of the SECM tip, Ir/Ta MMO, and fabricated crystalline $CoSb_xO_y$ electrodes in 5 M NaCl solution. For (a), only the Pt tip was biased. For (b)-(g), only the sample (fabricated electrodes or Ir/Ta MMO) was biased.

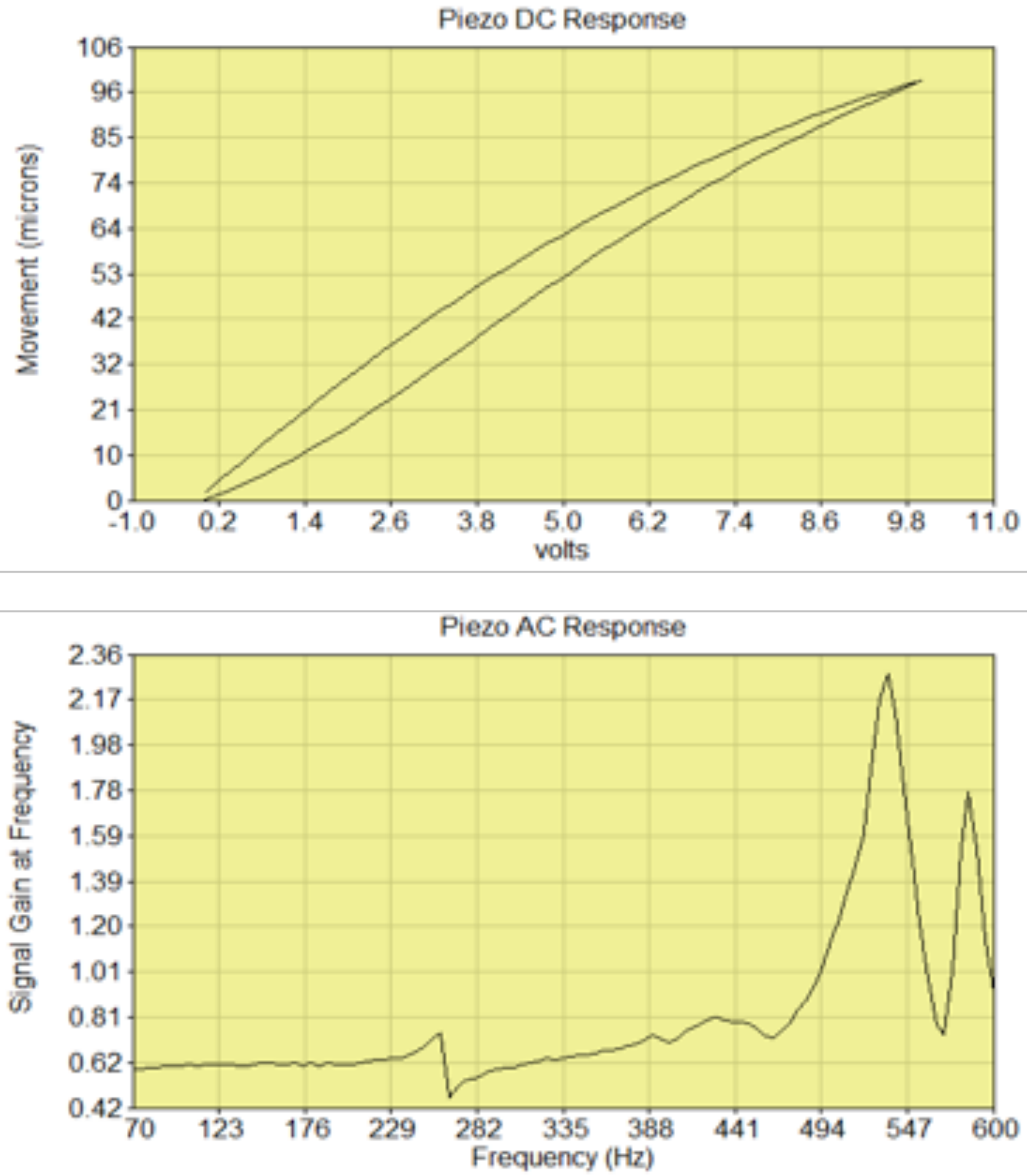

Figure S3. DC and AC response from a piezoelectric positioner to determine the resonance frequency. The resonance frequency was 540 Hz in this experiment.

In intermittent contact-scanning electrochemical microscopy (IC-SECM), the tip is controlled by a piezoelectric positioner in the z-direction (above the substrate), which is driven by an AC signal generator,

and intermittently contacts the substrate surface.[1] By monitoring the oscillation amplitude, which becomes dampened when the tip reaches the electrode surface (i.e., substrate surface of either Ir/Ta MMO or $CoSb_xO_y$), we determined the absolute z-position of the spot on the electrode directly under the tip.[1] By repeating this process at different spots on the electrode, IC-SECM can generate a topology map for the region of interest on the electrode. In this study, direct current (DC) and alternating current (AC) responses generated by piezoelectric control were collected to determine the resonance frequency (540 Hz) at which the tip sensitivity will be enhanced to detect the electrode surface and the vibration magnitude.

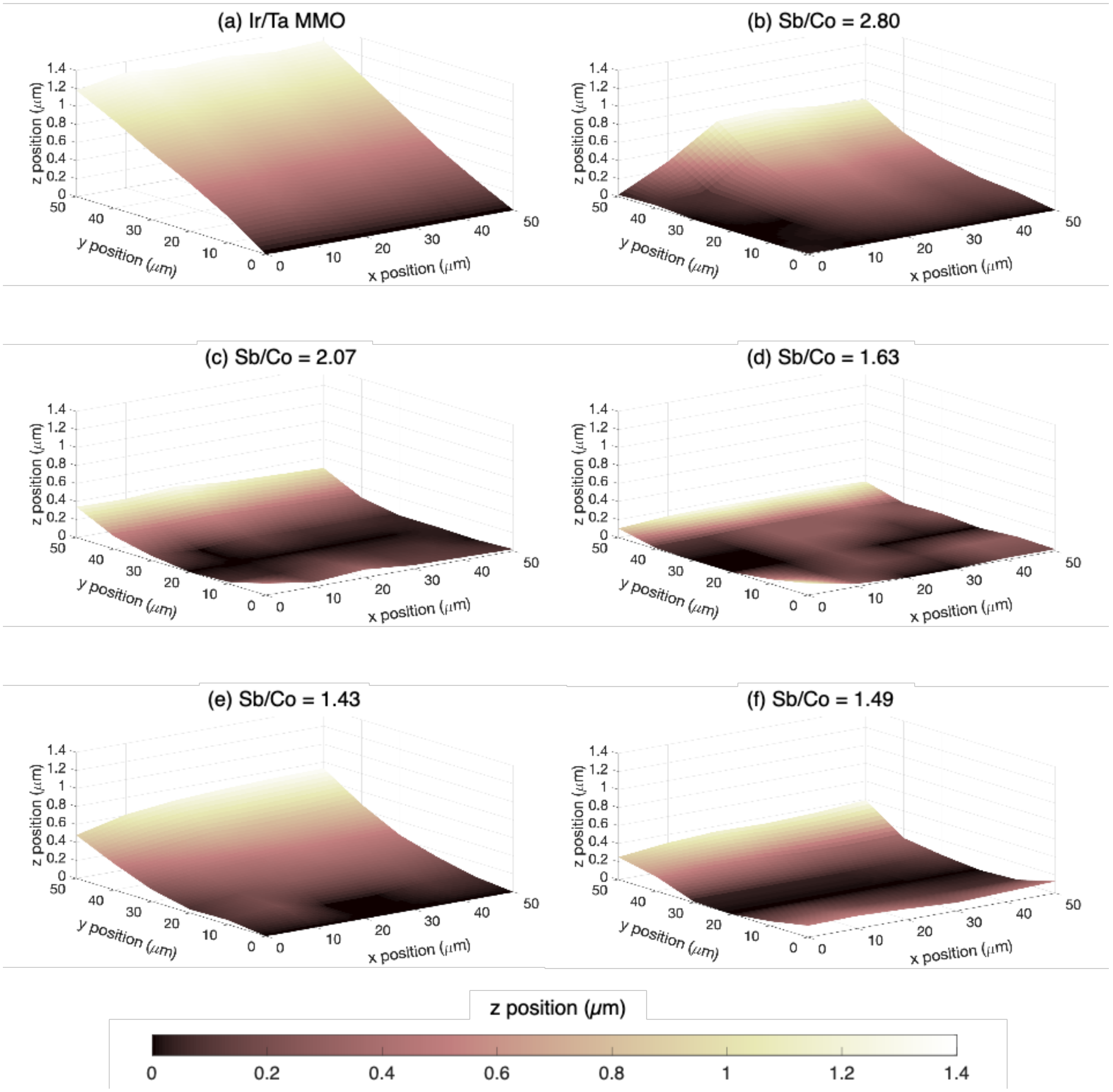

Figure S4. Topology of Ir/Ta MMO and fabricated crystalline $CoSb_xO_y$ electrodes acquired via IC-SECM.

Positioning the tip accurately is essential for any SECM measurement because measured current, the parameter that is directly related to electrochemical activities, changes with the tip-to-surface distance. Therefore, it is crucial to first precisely identify the absolute position of the electrode surface and the tip-to-surface distance with high spatial resolution. To decouple the contributions to measured current from

electrode topography and electrochemical activity, IC-SECM was employed. An operating frequency (525 Hz, 10-15 Hz lower than the resonance frequency) was chosen for the surface approach, or movement from bulk solution toward the sample-electrolyte interface, to measure the relative electrode surface position as $z = 0$ for the experiments. IC-SECM was performed within 1 μm tip-to-substrate distance to exclude the influence of sample topology on the measured current (surface slope < 1.5%).

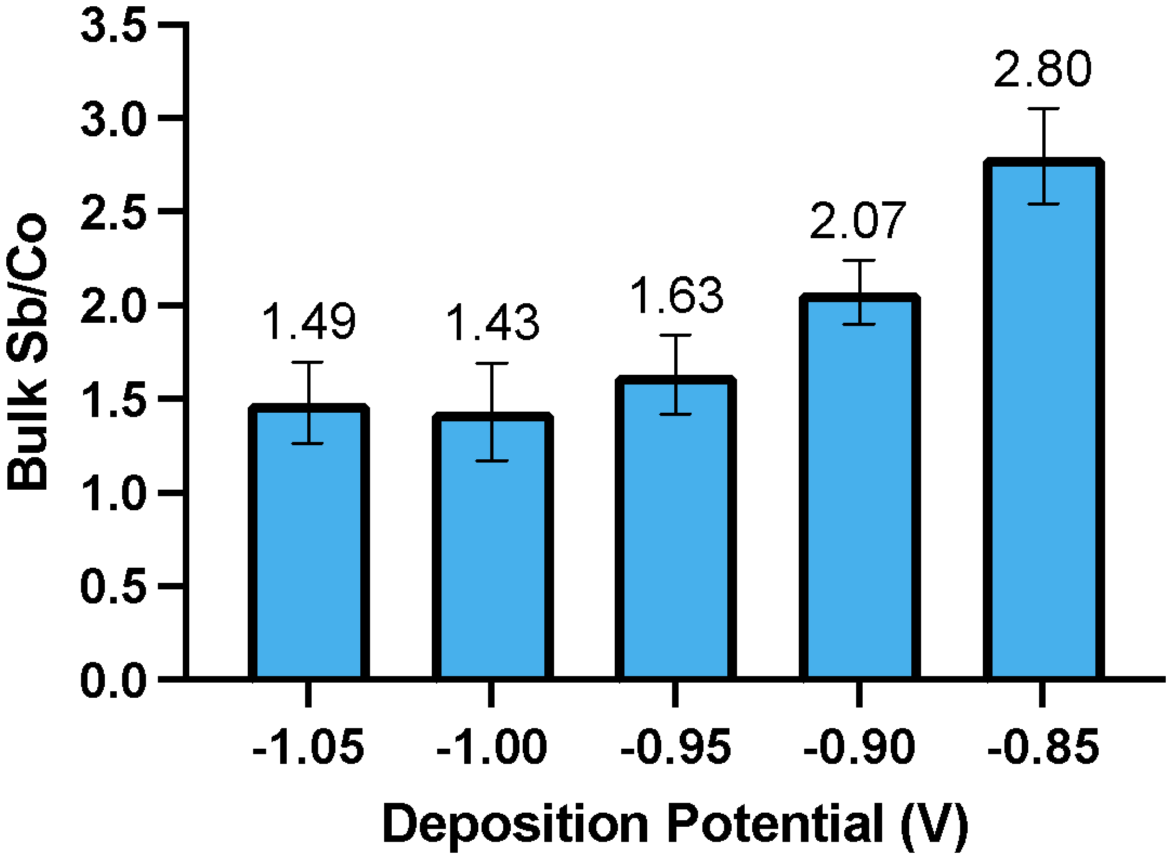

Figure S5. The bulk Sb/Co ratios for samples deposited at different potentials. The bulk Sb/Co ratios were obtained using EDS.

(a) Sb/Co = 2.80

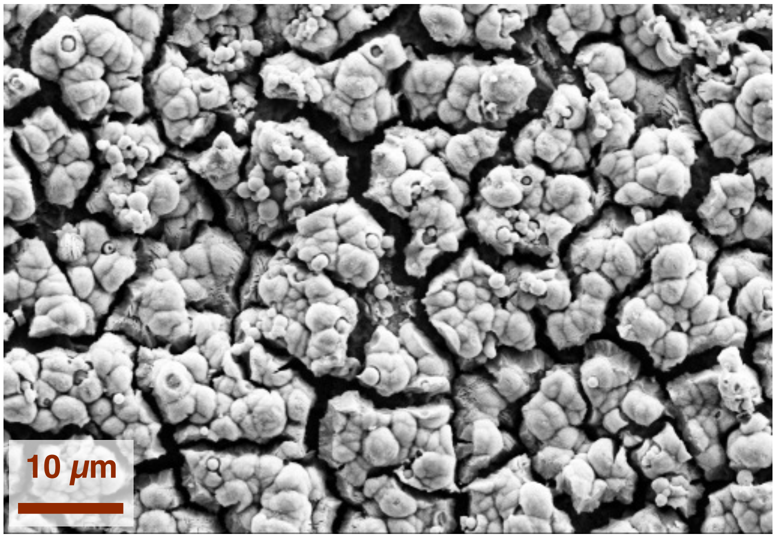

(b) Sb/Co = 2.07

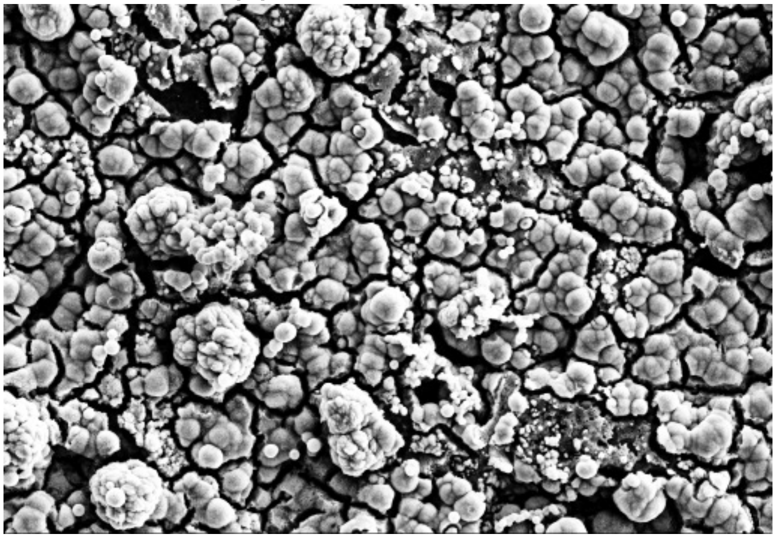

(c) Sb/Co = 1.63

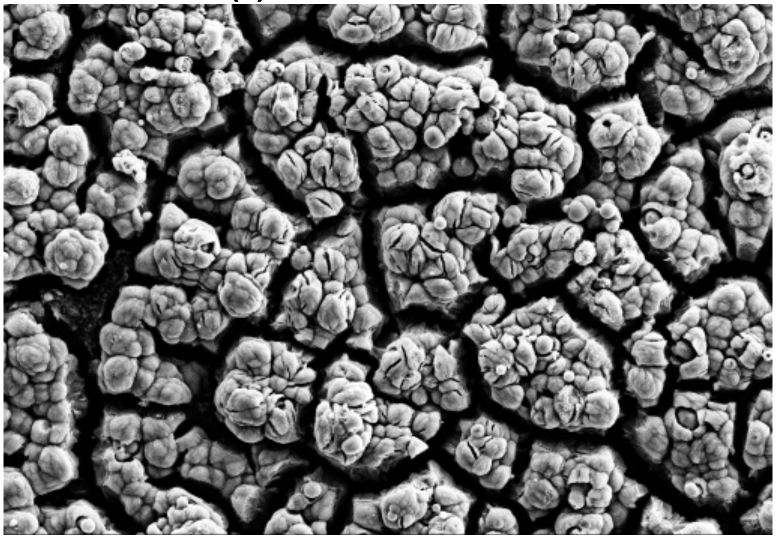

(d) Sb/Co = 1.43

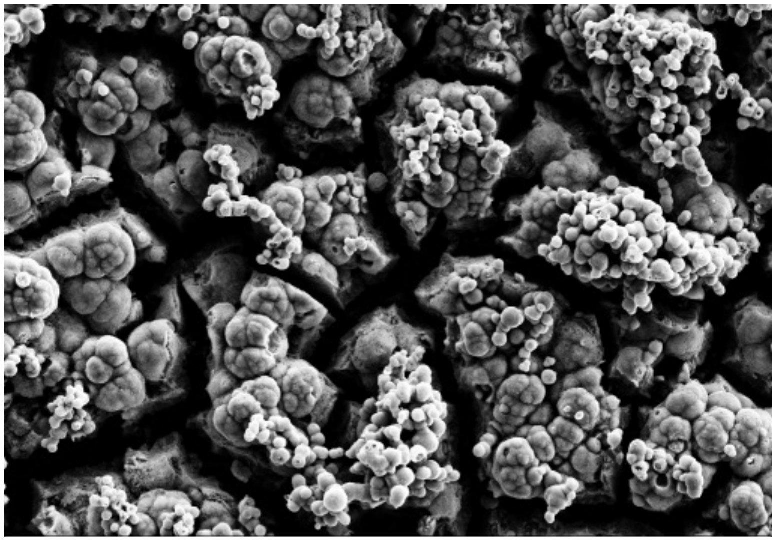

(e) Sb/Co = 1.49

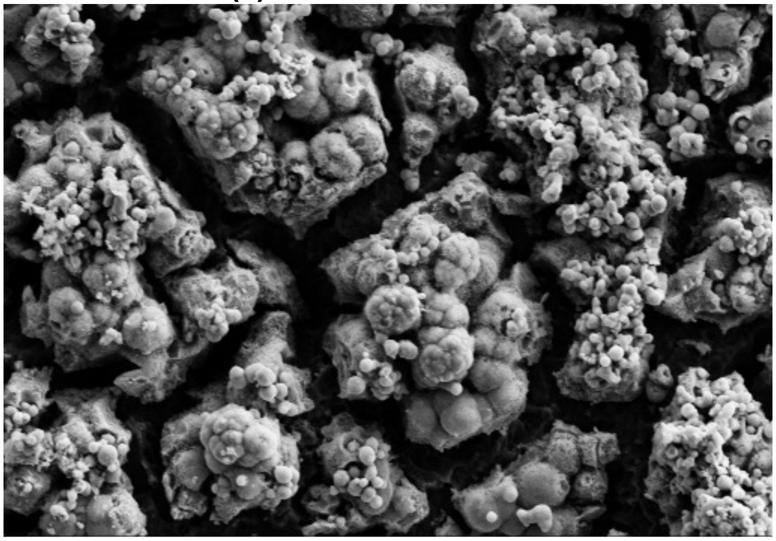

Figure S6. SEM images of samples that were derived from electrodeposited $Co(OH)_2/Sb$ films at (a) -0.85 V, (b) -0.90 V, (c) -0.95 V, (d) -1.00 V, and (e) -1.05 V. All potentials were versus saturated calomel electrode. Mud-crack morphology was observed on all samples; generally the lower the bulk Sb/Co ratios, the larger width the cracks.

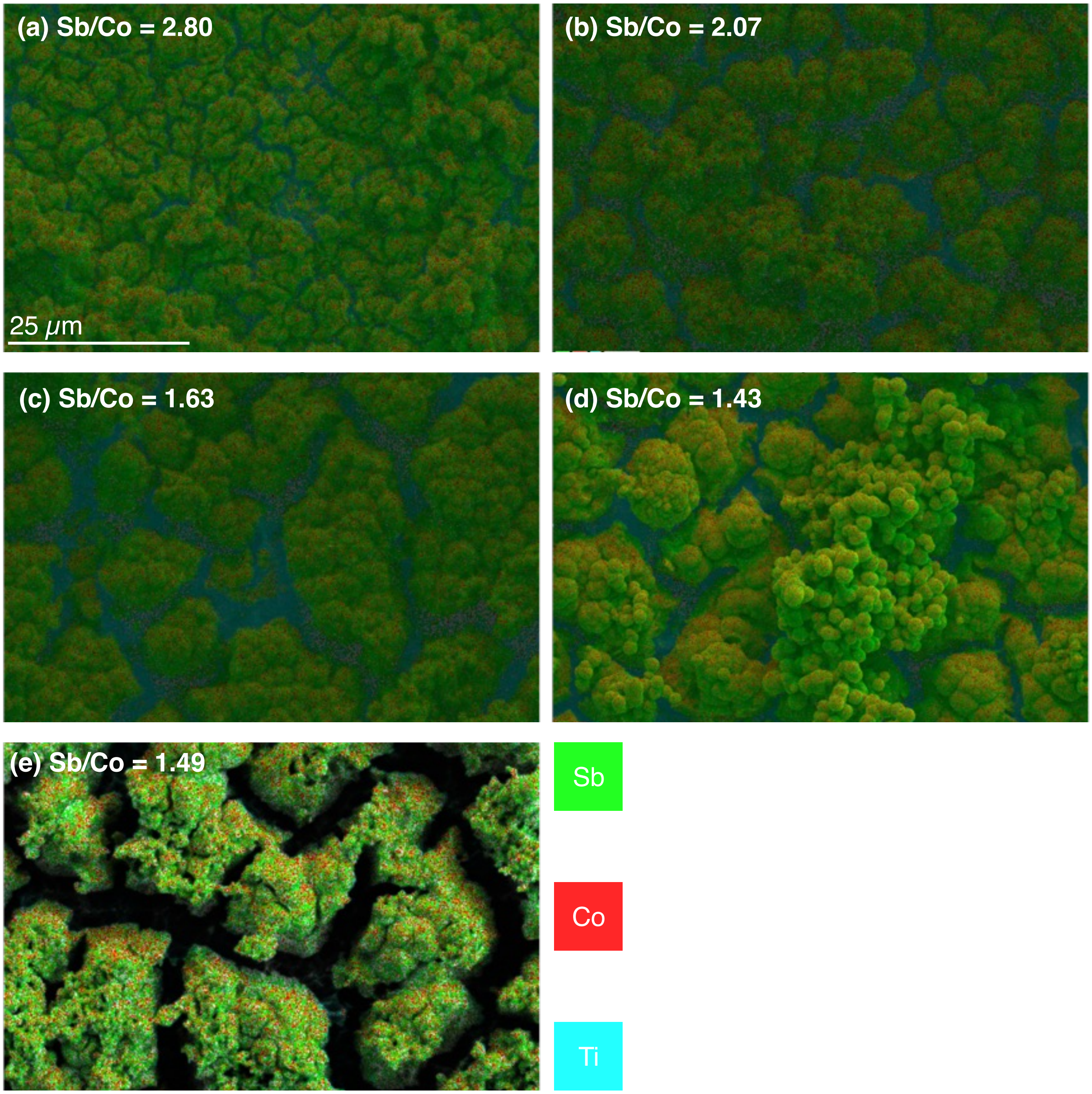

Figure S7. EDS mapping of the samples that were derived from electrodeposited $Co(OH)_2$/Sb films at (a) -0.85 V, (b) -0.90 V, (c) -0.95 V, (d) -1.00 V, and (e) -1.05 V. All potentials were versus saturated calomel electrode. Co and Ti were uniformly distributed in the samples.

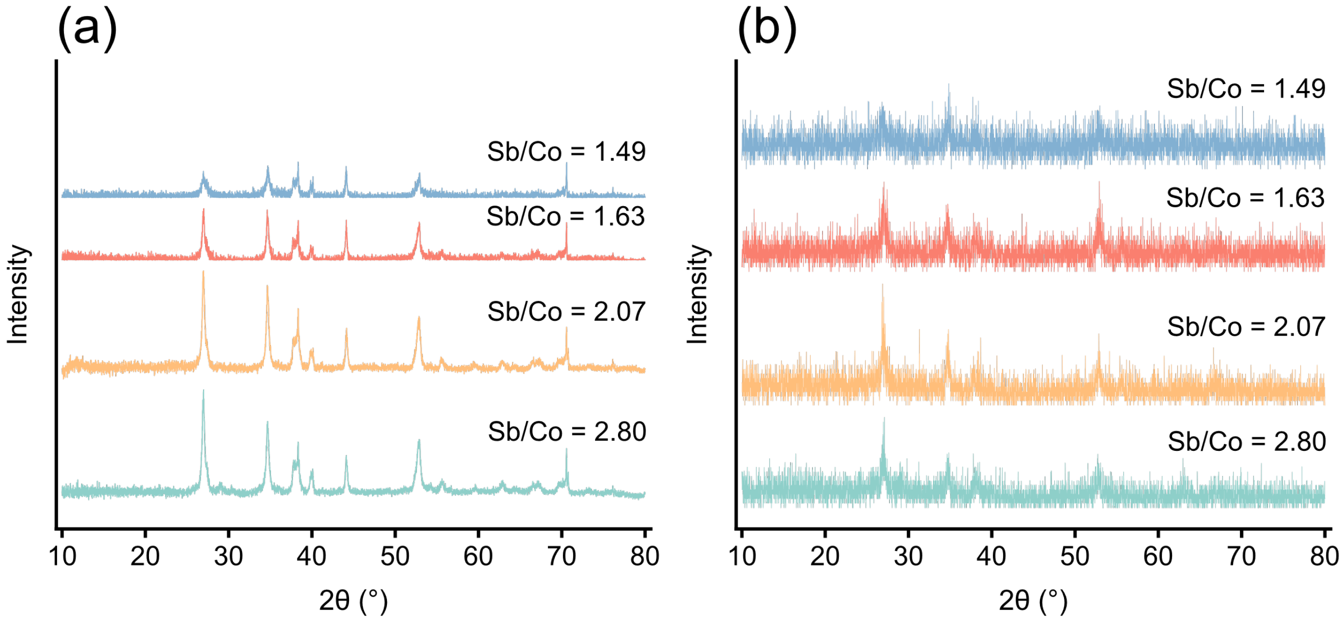

Figure S8. (a) bulk phase and (b) grazing incidence XRD for cobalt antimonate samples with various Sb/Co ratios. For GIXRD, the incidence angle was kept at 4° for all the samples.

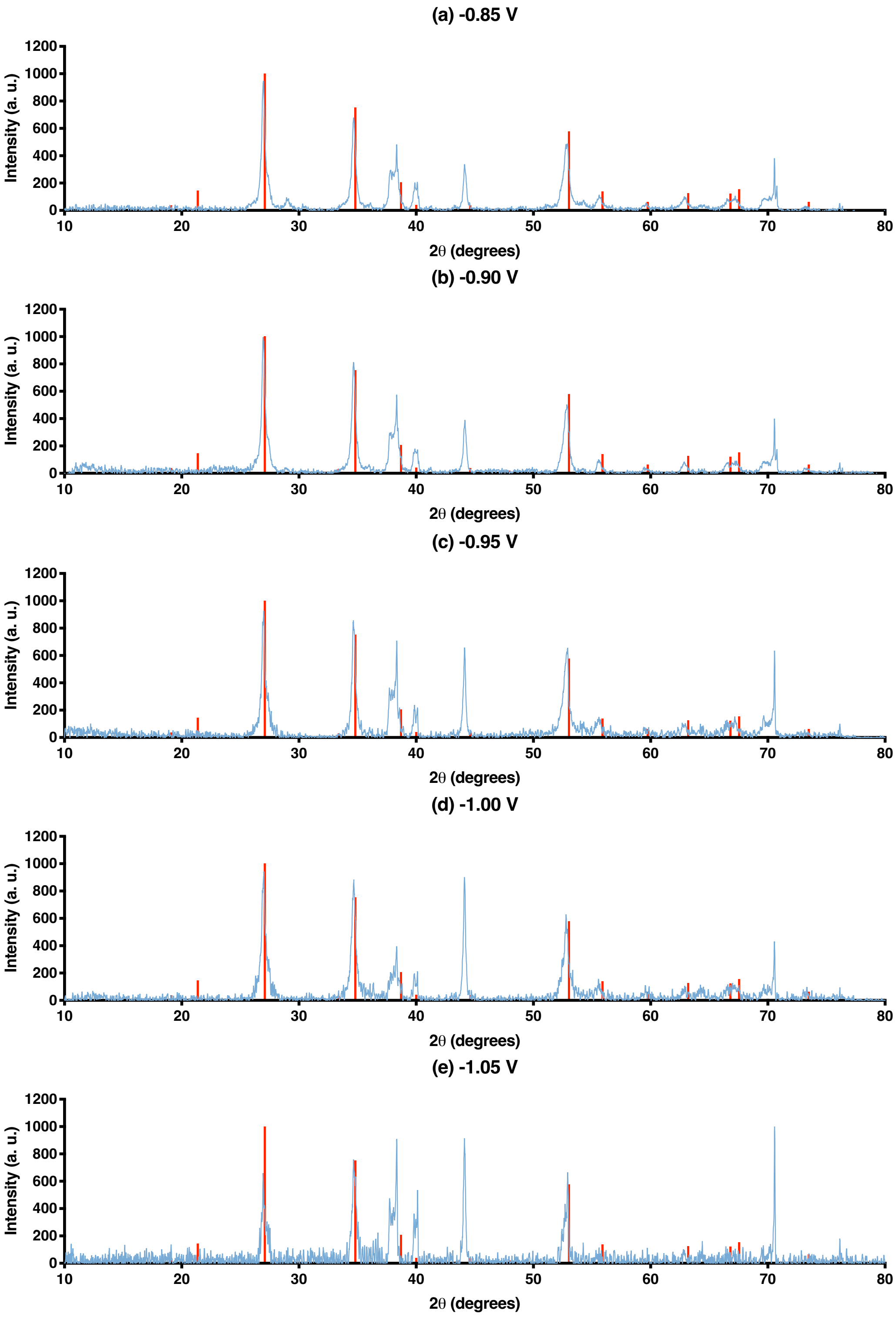

Figure S9. Experimental diffraction data for each electrode (e.g., deposition potentials: -0.85, -0.90, -0.95, -1.00, -1.05V v. SCE) versus the $CoSb_xO_y$ peaks and intensities stored in the Crystallography Open Database (COD). These XRD results were generated from a new set of electrode samples, which is

separated from the ones used to generate Figure S8 and in the electrochemical characterizations in the main manuscript.

Probable crystalline phases were determined using QualX2.0 in combination with COD.[2–8] QualX2.0 is a qualitative phase analysis software employed for identifying phases based on experimental diffraction data. The COD is an open-access repository containing various crystalline structures of organic, inorganic, and metal-organic compounds, as well as minerals.

QualX2.0 was utilized to identify experimental diffraction data for all potential crystalline phases that included cobalt, antimony, and oxygen. The search criteria were restricted to the species containing a single or a combination of Co, Sb, and O. The diffraction data interval ranged from 10 to 80 degrees. The threshold on intensity was set to 5.00 in order to eliminate interferences caused by background noise and accurately identify peaks of interest.

Search matching was employed to compare the COD reference patterns with the experimental diffraction data, enabling the identification of the most suitable matches. Following the search, QualX2.0 generated a sorted list of plausible phases based on their figure of merit (FoM). FoM is calculated by considering the number of matched peaks, the average distance in peak position, and the average difference in peak intensity between compounds in the COD and the experimental diffraction data.

Table S1. XRD peak characterizations.

	**$CoSb_xO_y$**			
Deposition Potential (*V v. SCE*)	Peak Position	Intensity	Scale	FoM
-0.850	0.68062	0.5253	0.53438	0.29503
-0.900	0.43376	0.54376	0.54398	0.23079

-0.950	0.44336	0.44017	0.41721	0.45134
-0.100	0.5145	0.41959	0.60976	0.43145
-0.105	0.6732	0.49892	0.58433	0.32669

The $CoSb_xO_y$ FoM varied across all electrodes, ranging between 0.23 and 0.45. With the FoM threshold set to 0.0, any crystalline phases identified with a calculated FoM below 0.0 were not considered to be plausible crystalline phases.

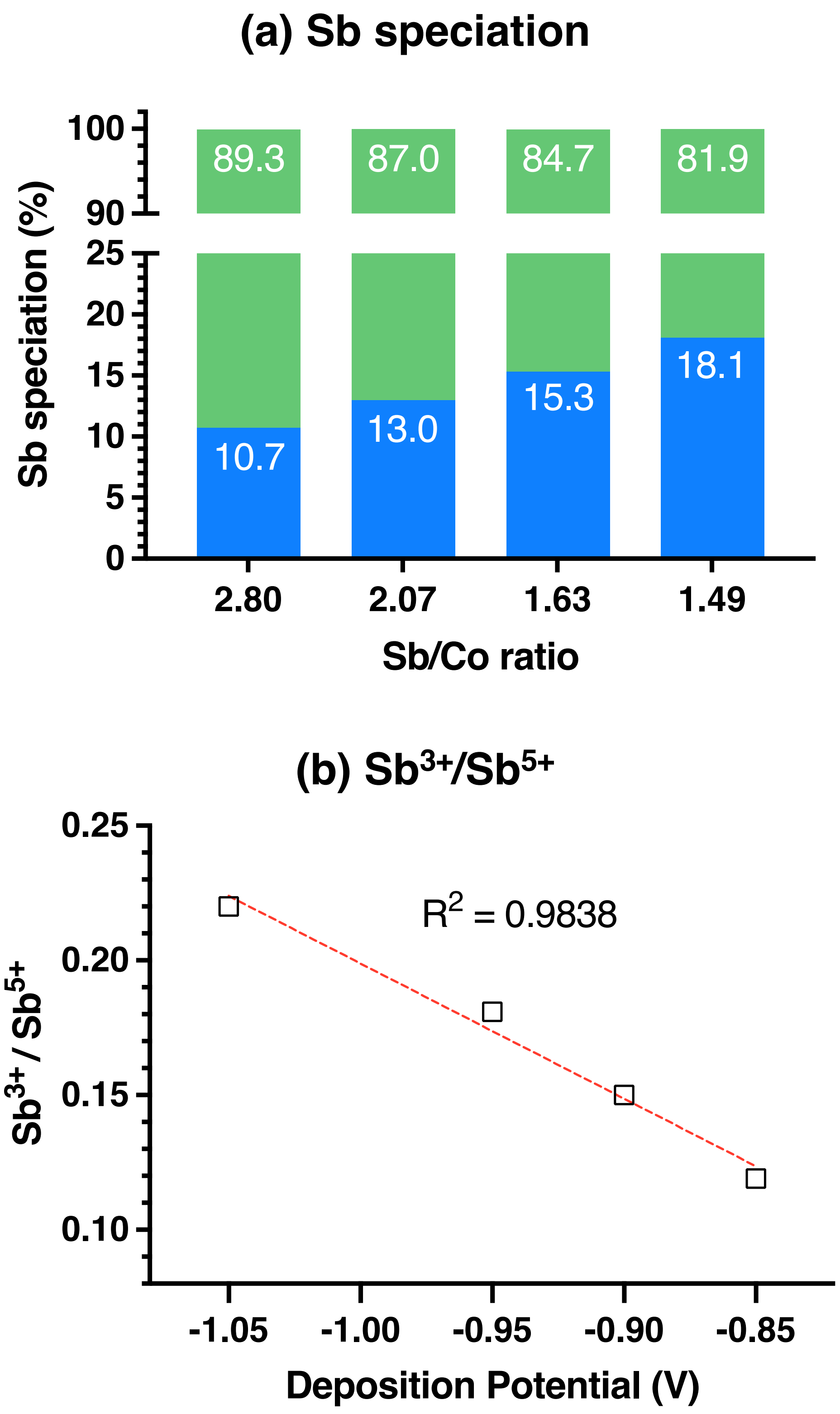

Figure S10. (a) percentage contribution of Sb^{3+} and Sb^{5+} in deconvoluted Sb $3d_{3/2}$ peaks with cobalt antimonate samples with different Sb/Co ratios. (b) Relationship between Sb^{3+}/Sb^{5+} and deposition potentials.

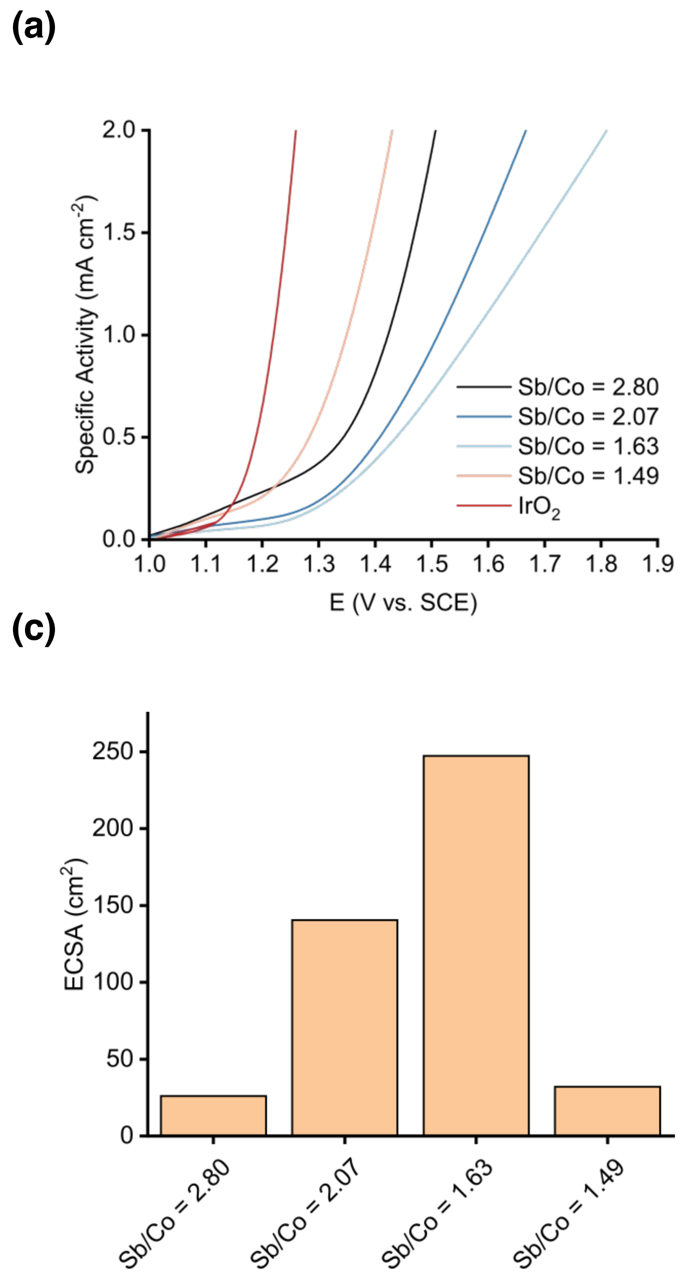

Figure S11. Specific activities of the different samples in (a) 5 M NaCl, and (b) 0.1 M H_2SO_4 solutions. To measure specific activities, linear sweep voltammetry was first conducted. At each potential step, the corresponding current was divided by (c) electrochemical surface area (ECSA) of samples with different Sb/Co ratios. The geometric surface areas of the samples were 2 cm^2.

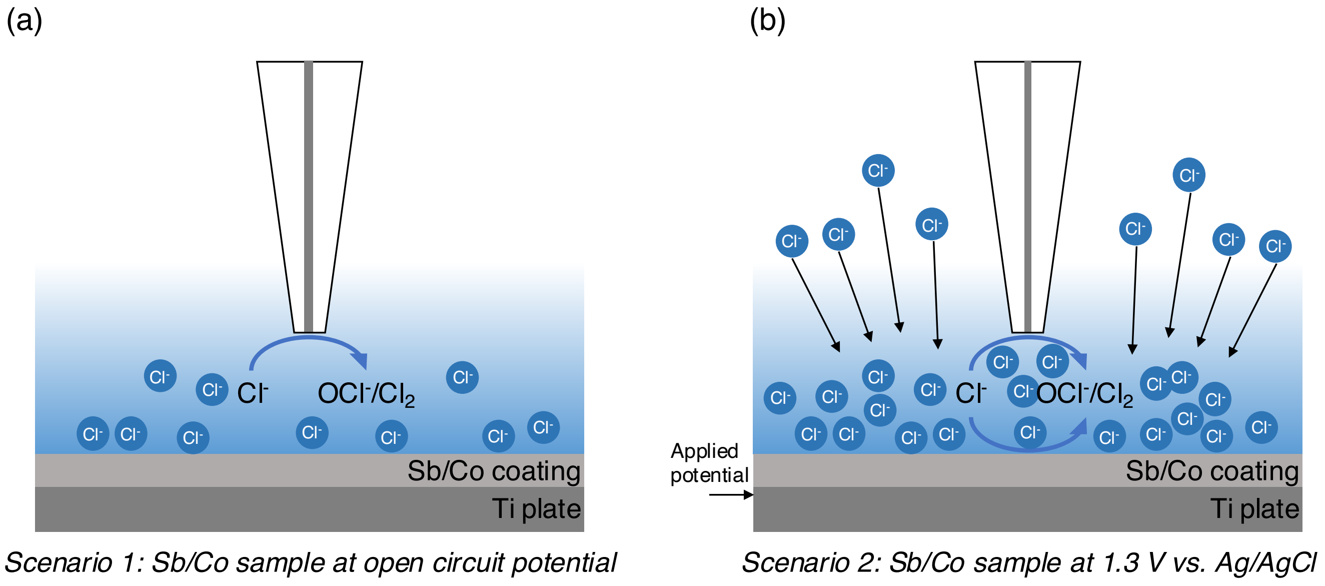

Figure S12. Two scenarios of SECM measurements. In scenario 1, the tip was biased at 1.4 V vs. Ag/AgCl, and the sample was at open circuit potential. In scenario 2, the tip was biased at 1.4 V vs. Ag/AgCl (as in scenario 1), and the sample was biased at 1.3 V vs. Ag/AgCl.

We posit that when the sample was biased in scenario 2, anion (i.e., chloride) was attracted towards the sample from the solution so that more chloride ions were present near the electrodes. Given the small distance between tip and sample (5 μm), the attracted chloride was also available for the tip to conduct chlorine evolution reaction (CER). This high concentration of reactant (i.e., chloride) could cause higher tip current in scenario 2 compared to in scenario 1, and therefore the percentage change of tip current was negative for some samples. For the samples that exhibited high activity towards CER, we infer that the sample consumed enough chloride during CER in scenario 2 so that the net change of the total amount of chloride ions available for CER at the tip did not increase, resulting in the negative percentage change in tip current.

References

(1) McKelvey, K.; Edwards, M. A.; Unwin, P. R. Intermittent Contact−Scanning Electrochemical Microscopy (IC−SECM): A New Approach for Tip Positioning and Simultaneous Imaging of Interfacial Topography and Activity. *Anal. Chem.* **2010**, *82* (15), 6334–6337. https://doi.org/10.1021/ac101099e.

(2) Downs, R. T.; Hall-Wallace, M. The American Mineralogist Crystal Structure Database. **2003**.

(3) Gražulis, S.; Daškevič, A.; Merkys, A.; Chateigner, D.; Lutterotti, L.; Quirós, M.; Serebryanaya, N. R.; Moeck, P.; Downs, R. T.; Le Bail, A. Crystallography Open Database (COD): An Open-Access Collection of Crystal Structures and Platform for World-Wide Collaboration. *Nucleic Acids Research* **2012**, *40* (D1), D420–D427. https://doi.org/10.1093/nar/gkr900.

(4) Merkys, A.; Vaitkus, A.; Butkus, J.; Okulič-Kazarinas, M.; Kairys, V.; Gražulis, S. COD::CIF::Parser: An Error-Correcting CIF Parser for the Perl Language. *J Appl Cryst* **2016**, *49* (1), 292–301. https://doi.org/10.1107/S1600576715022396.

(5) Gražulis, S.; Merkys, A.; Vaitkus, A.; Okulič-Kazarinas, M. Computing Stoichiometric Molecular Composition from Crystal Structures. *J Appl Cryst* **2015**, *48* (1), 85–91. https://doi.org/10.1107/S1600576714025904.

(6) Quirós, M.; Gražulis, S.; Girdzijauskaitė, S.; Merkys, A.; Vaitkus, A. Using SMILES Strings for the Description of Chemical Connectivity in the Crystallography Open Database. *Journal of Cheminformatics* **2018**, *10* (1), 23. https://doi.org/10.1186/s13321-018-0279-6.

(7) Vaitkus, A.; Merkys, A.; Gražulis, S. Validation of the Crystallography Open Database Using the Crystallographic Information Framework. *J Appl Cryst* **2021**, *54* (2), 661–672. https://doi.org/10.1107/S1600576720016532.

(8) Merkys, A.; Vaitkus, A.; Grybauskas, A.; Konovalovas, A.; Quirós, M.; Gražulis, S. Graph Isomorphism-Based Algorithm for Cross-Checking Chemical and Crystallographic Descriptions. *Journal of Cheminformatics* **2023**, *15* (1), 25. https://doi.org/10.1186/s13321-023-00692-1.
